# Supplementary material for: Herpes Simplex Virus Type 1–Encoded miR-H2-3p Manipulates Cytosolic DNA–Stimulated Antiviral Innate Immune Response by Targeting DDX41
Source: Viruses. 2019 Aug 15;11(8):756. doi: 10.3390/v11080756 (PMC6723821; doi:10.3390/v11080756)
Supplement: Supplementary file 1 [file viruses-11-00756-s001.pdf]

Table S1. The prediction of HSV-1 miRNAs targeting DDX41 by RNAhybrid

Target sites<br>miRNAs	Predicted consequential pairing of target region (top)<br>and miRNAs (bottom)	MFE<br>(kcal/mol)
DDX41(96-101)<br>miR-H2-3p	5' AGCAUUAUC**GCUCAG**C 3'<br>3' CAGCGUGAGCAGGGCA**CGAGUC**C 5'	-20.2
DDX41(125-130)<br>miR-H13	5' AGCUGGCCUGGAAUGGGCCAGGCUG**GUCCUG**G 3'<br>3'GUCACGAGCGUGAAG**CGGGAU**U 5'	-20. 9
DDX41(175-181)<br>miR-H6-5p	5'UACUGUUUUUGUUU**CCUUUUACC**C 3'<br>3'UGUGUGGGGGGGACAC**GGAAGGUGG** 5'	-21.2
DDX41(113-119)<br>miR-H26	5' AGCUCAGCUGGCCUGGAA**UGGGCCA**G 3'<br>3' UGGCAGCGAGUG**GCUCGGU** 5'	-21.8
DDX41(125-131)<br>miR-H3-3p	5' GCUCAGCUGGCCUGGAAUGGGCCAGGC**UGGUCCUGG**C 3'<br>3' AGGGUUGGCGU**GUCAGGGUC** 5'	-22.3
DDX41(138-144)<br>miR-H5-5p	5' UGGCUGCCU**GUUCCCU**G 3'<br>3' AUCUCUACGGGCU**UGGGGGG**G 5'	-22.6
DDX41(19-24)<br>miR-H29	5' CAGUCUUCCCUUC**UCUCCA**A 3'<br>3' CAUCAGGAACGGGC**GGAGGU**C 5'	-23.5
DDX41(70-76)<br>miR-H5-5p	5'AAGACUGCCACCAGUCUACACAUACAGC**AGCCCCCU**G 3'<br>3'AUCUCUACGGGC**UUGGGGGG**G 5'	-24.7
DDX41(149-155)<br>miR-H29	5' CUGGUCCUGGCUGCCUGUUCCCUGUGC**UCUUCAG**A 3'<br>3' CAUCAGGAACGGGC**GGAGGUC** 5'	-29.6

Nucleotides in bold fonts show the seed sequence of miRNAs matching DDX41 mRNA sequence.

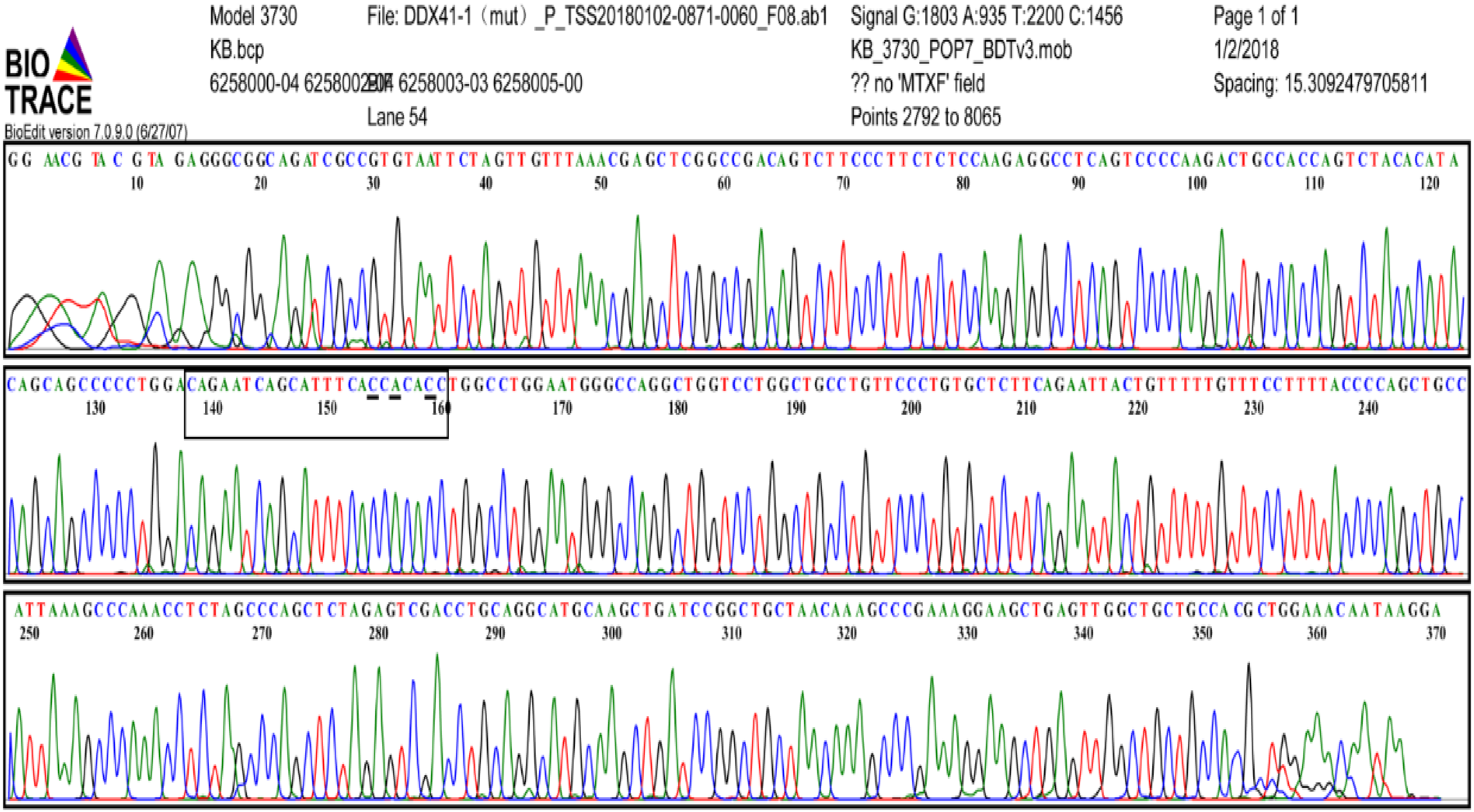

Figure S1. The sequencing results of pmirGLO-DDX41 site-directed mutation plasmid

The black box shows the sequence matching with miR-H2-3p in paper, the black underline of nucleotides shows the mutation sites.

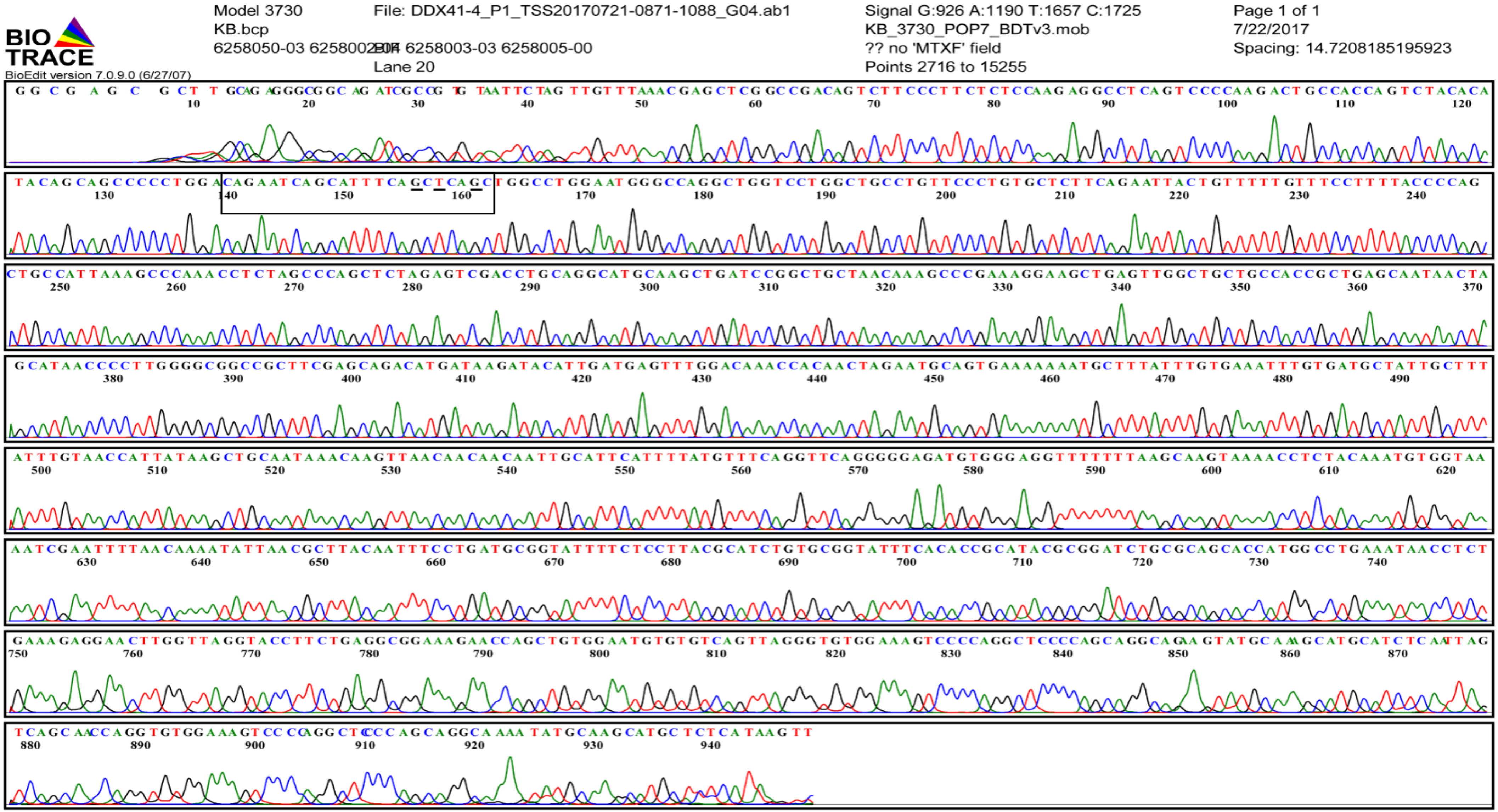

Figure S2. The sequencing results of pmirGLO-DDX41 wild type plasmid
